# High-viscosity α-starch nanogel particles to enhance oil recovery

**DOI:** 10.1039/c9ra06938k

**Published:** 2020-02-26

**Authors:** Tuo Liang, Jirui Hou, Ming Qu, Mengdan Zhao, Infant Raj

**Affiliations:** China University of Petroleum-Beijing Changping 102249 Beijing P. R. China m.qu@foxmail.com

## Abstract

The formation of dominant water channels is a serious problem for most oilfields, which results in low sweep efficiency. Recently, gels regarded as materials for the conformance improvement of water have attracted significant attention for increasing the sweep efficiency in many reservoirs suffering from water invasion but no effect on oil displacement efficiency. Nanogel particles possessing synergic properties that increase sweep efficiency and oil displacement efficiency have not been previously reported. Herein, economical high-viscosity α-starch nanogel particles were synthesized through a free radical reaction to play the synergistic role of gel and nanoparticles. The average diameter of the nanogel particles was 30 nm with a dispersion viscosity of 250 mPa s at 90 °C. A linear formula describing the relationship among the nanogel particle dispersion viscosity, temperature and concentration was also perfectly fitted. Core flooding experiments have demonstrated that both light and heavy oil recovery rates reached around 30%. The EOR mechanisms and flow behaviors of the nanogel particles were revealed through 2-D visualized model experiments under different conditions. On the one hand, nanogel particles could displace oil droplets from the rock surface due to the creation of the structural disjoining pressure. On the other hand, nanogel particle dispersion with high viscosity could increase the sweep efficiency and drag oil clusters out of the oil phase. Therefore, nanogel particles could be regarded as a potential candidate for enhancing oil recovery.

## Abstract 2

The formation of dominant water channels is a serious problem for most oilfields, which results in low sweep efficiency.

## Graphical abstract

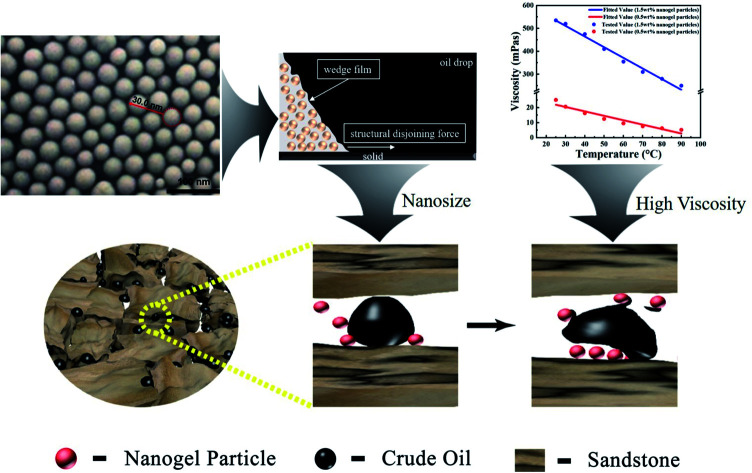

## 1 Introduction

In recent years, there has been a serious issue concerning the dramatically increasing oil demands and decreasing oil production caused by the majority of mature oilfields entering a high water cut period.^1–3^ Recently, new materials and technologies for controlling water conformance in the oilfields to prevent water from production wells and increase oil production of remaining oil after water flooding were explored.^4–6^

Different kinds of materials have been employed to plug water channels and reduce high water cut to enhance oil recovery. One of the methods, namely gel treatment, has attracted significant attention from researchers for the reduction of water production as a feasible and economical technology.^7^ The gel treatment not only improved the viscosity of the displacement phase but also increased the microscopic sweep efficiency by plugging highly permeable zones. There are two common kinds of gel: *in situ* bulk gel and prepared gel.^8–11^

A mixture of crosslinker and polymer solution is injected into formation first, and then the gel is formed at *in situ* reservoir conditions to plug water channels. Oil production was achieved by utilizing *in situ* gel to control water conformance and increase sweep efficiency.^12,13^ However, several drawbacks restricted *in situ* bulk gel application in some harsh reservoirs, such as the lack of gel strength control, gelation time control, adsorption water and chromatographic fractionation.^1,10,14,15^

Some researchers have proposed a new type of gel synthesized before water injection to overcome the disadvantages of *in situ* bulk gel. This novel gel has better performance in complex reservoir conditions such as high temperature and salinity.^4,10^ Hence, many researchers have been interested in synthesizing various novel gels including PPGs, microgel particles and pH-sensitive polymer microgels. PPGs are superabsorbent crosslinking polymers with micro- to millimeter-scale sizes.^4,16^ Microgel particles are studied due to their stability, elastic deformation and size-control. The size of the microgel particles was 1000 nm less than that of PPGs.^17,18^ The pH-sensitive polymer microgel, as the initiator, was synthesized by altering the pH. It had excellent stability under the maximum pressure gradients of 4000 psi per ft.^19,20^ The main differences in the above gels were particle size, swelling ratio and gel strength.

However, these gels mentioned above were able to improve the sweep efficiency but had a limited effect on improving the oil displacement efficiency. The nanomaterial technique, as a new technology for further effectively improving oil displacement efficiency, has been widely studied.^21–23^ There are many published papers concentrated on interfacial tension changes, emulsion stabilization and foams from nanoparticle absorption at interfaces.^24–26^ Moghadam and Azizian^27^ investigated the synergistic effect of ZnO nanoparticles and cationic surfactant CTAB on the dynamic and equilibrium interfacial tension. The results showed that nanoparticles in the presence of CTAB decreased the interfacial tension by a synergistic effect, while the nanoparticles solely had no prominent contribution to the reduction of interfacial tension. Experiments regarding the effects of hydrophilic silica nanoparticles in the presence of cationic surfactants on the interfacial tension were also conducted. The results illustrated that the silica nanoparticles increased the interfacial tension.^28^ Ma *et al.*^29^ researched the influence of silica nanoparticles on surface and interfacial tensions in the presence of anionic and non-ionic surfactants; their results showed that interfacial tension decreased with the addition of silica nanoparticles.^29^ The nanomaterials have excellent potential to improve the efficiency of oil displacement but have limited effects on improving the sweep efficiency. To date, there has been no work on simultaneously utilizing materials possessing both the properties of nanoparticles and gel.

In this study, in order to simultaneously determine the synergistic effects of gel and nanomaterials on enhanced oil recovery, we synthesized novel nanogel particles with a diameter of 30 nm through a free radical reaction using α-starch, acrylamide, *N*,*N*′-methylene bisacrylamide, and potassium persulfate. The viscosity of the nanogel particle dispersion was also measured under different conditions using a viscometer. Core flooding experiments were conducted to test the effects of the nanogel particles when used for enhanced oil recovery under different driving conditions. Moreover, several complex 2-D visualized models were also designed and fabricated to study the mechanisms and flow behaviors of the nanogel particles during the process of oil displacement.

## 2 Methods

### 2.1 Synthesis of nanogel particles

Nanogel particles used in this study were synthesized by a free radical reaction using α-starch, acrylamide, *N*,*N*′-methylene bisacrylamide (crosslinking agent), potassium persulfate (initiator), and deionized (DI) water.

**Fig. 1:**
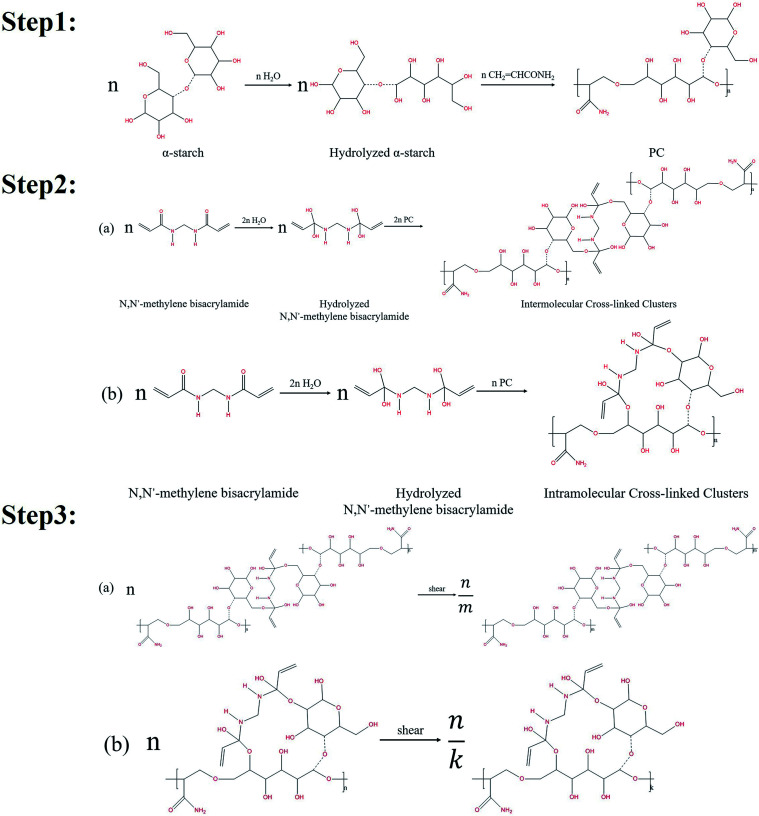
Gelation mechanisms of the cross-linking reaction between the α-starch and *N*,*N*′-methylene bisacrylamide.

The organic cross-linking reactions of the *in situ* α-starch gel systems are related to the hydroxyl groups (–CH_2_OH). Fig. 1 illustrates the reaction steps in a complete α-starch-based gel system.

Firstly, the ether bond of one six-membered ring in the α-starch molecule was opened by hydrolysis to form two hydroxyl groups. Then, with the effect of the initiator, the hydrolyzed α-starch reacted with acrylamide to facilitate the polymer condensation (PC), which is shown in step 1 of Fig. 1. Secondly, the carbonyl of the *N*,*N*′-methylene bisacrylamide molecule was converted into hydroxyl groups by hydrolysis. PC was further carried out *via* the hydroxyl dehydration condensation with hydrolyzed *N*,*N*′-methylene bisacrylamide to form intermolecular/intramolecular cross-linked clusters (several different structures, as shown in step 2 of Fig. 1). Thirdly, the chain length of the intermolecular/intramolecular clusters decreased to facilitate macromolecular agglomeration under mechanical shearing (only one of the structures is indicated), which is shown in step 3 of Fig. 1. Notably, there are lots of hydroxyl groups exposed in macromolecular agglomerations, providing more opportunities for cross-linking to form high-viscosity nanogel particles.

**Fig. 2:**
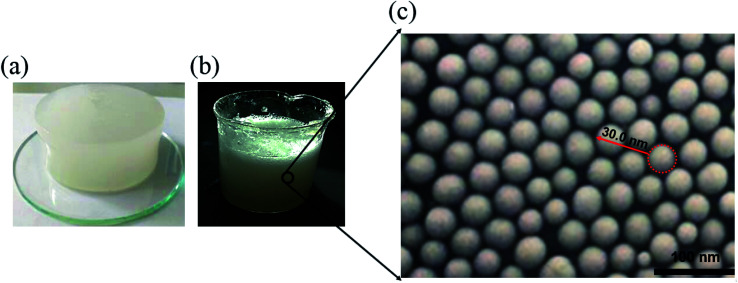
(a–c) α-Starch gel, nanogel particle dispersion, and SEM image of the nanogel particles, respectively.

Based on the above chemical reactions, the aqueous solution containing 3 wt% α-starch, 3 wt% acrylamide, 0.15 wt% *N*,*N*′-methylene bisacrylamide, 0.2 wt% potassium persulfate was allowed to stand for 30 minutes at 50 °C to gel (Fig. 2(a)). Afterwards, the nanogel particle dispersion was obtained by stirring the gel at 2000 rpm for 1 minute (Fig. 2(b)).

### 2.2 Chemicals used

The oil for the 2-D visualized model experiments was a mixture of aqueous kerosene and paraffin with the volume ration of 1 : 20. The viscosity was 23.9 mPa s at 25 °C, measured using a HAAKE RS600 rheometer. Sudan III was added to the oil mixture to enhance the visual effect, turning the color of the model oil into red.

The oil for the core flooding experiments was provided by the Daqing oilfield. The viscosity was 25 mPa s and 100 mPa s at 25 °C, measured by a HAAKE RS600 rheometer.

The Daqing oilfield synthetic brine (Table 1) was employed to conduct the 2-D visualized model and core flooding experiments. Additionally, the brine was dyed blue in 2-D visualized experiments for better visual effects by adding 0.05 mg L^−1^ of methylene blue.

**Table 1:** Ion compositions of simulated formation water

Concentration of ions (mg L^−1^)							Total salinity (mg L^−1^)
Ca^2+^	Mg^2+^	K^+^ + Na^+^	CO_3_^2−^	HCO_3_^−^	Cl^−^	SO_4_^2−^	
15	7	2428	198	2160	2267	54	7129

### 2.3 Viscosity measurement

The viscosities of the nanogel particles, at different concentrations (0.5 wt% and 1.5 wt%) *versus* different temperatures ranging from 25 °C to 90 °C, were measured by the HAAKE RS600 rheometer (Thermo Electron Co., Germany).

### 2.4 Cores for flooding experiments

Reservoir sandstones of the Daqing oilfield were used for flooding experiments with various permeabilities ranging from 25 mD to 2500 mD. Additionally, the cores were cylindrical with a diameter of 25 mm and a length of 100 mm. The processes of the core flooding experiments are as follows:

① The cylindrical core was placed in a core holder and after confining pressure was added to the core, the air was removed from the fixed core by a vacuum pump.

② The core without air was saturated with simulated formation water by a constant flow pump at 1 mL min^−1^. The volume of saturated water was recorded as *V*_wi_, representing the volume of pore space (*V*_p_).

③ The crude oil was injected to displace the simulated formation water at 0.2 mL min^−1^ using a constant flow pump. The volume of displaced water was recorded as *V*_oi_ to calculate the initial oil saturation (*S*_oi_) according to eqn (1). Additionally, the prepared core was aged for 3 days to simulate the formation of actual reservoirs.1
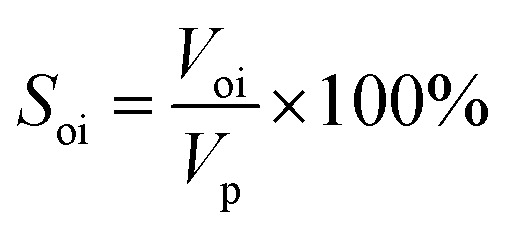


④ Water flooding was performed at 0.2 mL min^−1^ until the water cut reached 98%, and the oil production (*V*_ow_) and oil recovery rate (*R*_ow_) were calculated according to eqn (2). The nanogel particle dispersion was injected at the same velocity of 0.2 mL min^−1^. The secondary oil production (*V*_op_) and oil recovery rate (*R*_op_) were calculated using eqn (3).

⑤ The above steps were repeated when other parameters were investigated.2
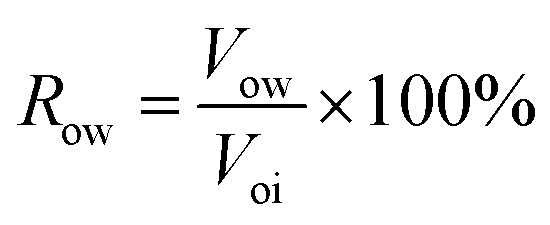
3
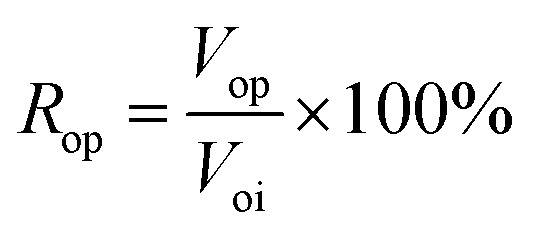


### 2.5 2-D visualized models

**Fig. 3:**
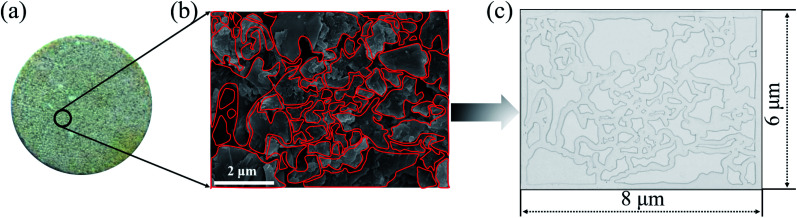
The procedure for making the 2-D visualized model with a permeability of 25 mD. (a)–(c) The natural core tablet, SEM image and diagram of the core tablet, 2-D visualized model derived from the SEM diagram, respectively.

**Fig. 4:**
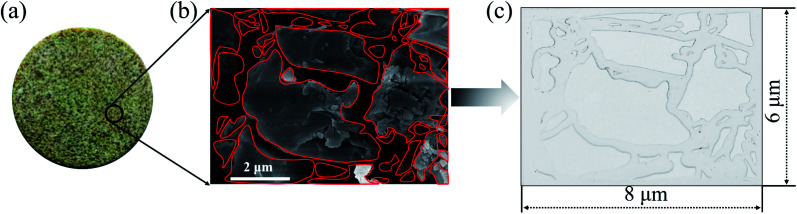
The procedure for making the 2-D visualized model with a permeability of 2500 mD. (a)–(c) The natural core tablet, SEM image and diagram of the core tablet, 2-D visualized model derived from the SEM diagram, respectively.

The 2-D visualized models were designed and fabricated based on the scanning electron microscope (SEM) investigation of core tablets. It was difficult to describe all the characteristics of totally different pores and throats of natural core tablets (Fig. 3(a) and 4(a)) derived from the Daqing oilfield. Thus, the 2-D visualized model diagrams were designed based on SEM images (Fig. 3(b) and 4(b)). For better visual effects, the 2-D visualized models were made of oil-wet transparent polymethyl methacrylate. A laser numerical control lathe was utilized to engrave the inner pores and throats. Finally, the 2-D visualized models, with permeabilities of 25 mD (Fig. 3(c)) and 2500 mD (Fig. 4(c)), were obtained to conduct experiments to determine the EOR mechanisms and flow behaviors of the nanogel particles in porous media.

## 3 Results and discussion

### 3.1 Properties of the nanogel particle dispersion

#### 3.1.1 Diameter of the nanogel particles

SEM images of the nanogel particles were obtained in order to assess their diameters. As shown in Fig. 2(c), the average diameter of the nanogel particles was about 30 nm, which is much smaller as compared to the conventional preformed gel particles.^30–32^ The smaller the particle size, the easier it is to get into the micropore throats, which is beneficial for enhancing oil recovery. Moreover, the nanogel particles were also uniformly distributed in solution, leading to excellent stability.

#### 3.1.2 Viscosity of the nanogel particle dispersion

**Fig. 5:**
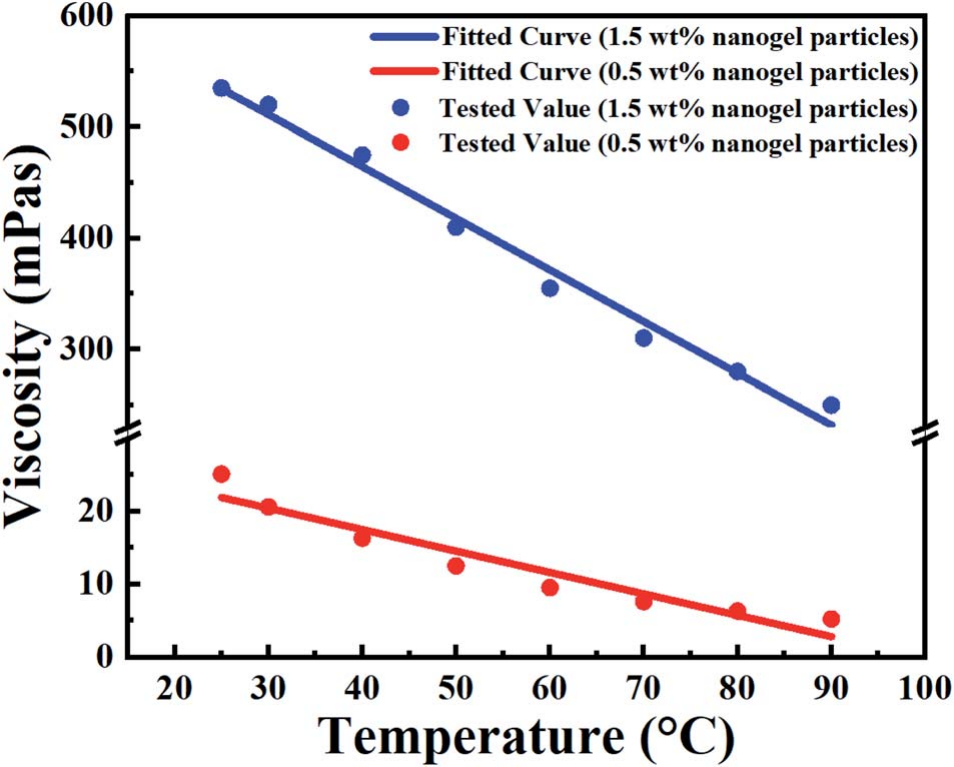
Fitted (curves) and measured (points) viscosity of the nanogel particle dispersion *versus* the temperature and concentration of nanogel particles.

The viscosity of the nanogel particle dispersion as a function of temperature and concentration is shown in Fig. 5 (points).

In terms of temperature, the viscosity of the nanogel particle dispersion gradually decreased with increasing system temperature for both 1.5 wt% and 0.5 wt% concentrations of nanogel particles. However, the viscosity for the 1.5 wt% nanogel particle system decreased faster than that of the 0.5 wt% nanogel particle system. This may be explained by the fact that the high-viscosity system was more sensitive to temperature. It is noteworthy that there was a linear relationship between temperature and viscosity. The different linear relationships were fitted and are exhibited in Table 2.

**Table 2:** The linear relationships between the viscosity and temperature of the nanogel particle dispersion under different concentrations of nanogel particles^a^

Concentration of nanogel particles (wt%)	Linear relationship	Adj. *R*-square
0.5	*η* = −0.29403 × *T* + 29.24278	0.92378
1.5	*η* = −4.65133 × *T* + 650.50533	0.98728

a Where *η* is the viscosity of the nanogel particle dispersion (mPa s); *T* is the ambient temperature (°C).

The correlations were trustworthy according to the value of Adj. *R*-square representing the degree of fitting.

Moreover, the viscosity of the nanogel particle dispersion dramatically increased on increasing the concentration of the nanogel particles and the viscosity was much higher than that of B-PPG.^31^ The high viscosity of the dispersion for 1.5 wt% nanogel particles was attributed to stronger intermolecular entanglement and the formation of three-dimensional complex network structures.^31^

Based on a detailed analysis of the relationships among the viscosity of nanogel, particle dispersion, temperature and concentration of nanogel particles, a comprehensive correlation (eqn (4)) was obtained and utilized to describe all the experimental data. The fitted curves are shown in Fig. 5 and the values are consistent with the experimental data.

4 *η*(*α*,*T*) = (*A* × *T* + *B*) × *α* + *C* × *T* + *D*

where *α* is the concentration of the nanogel particles (mg L^−1^); *A*, *B*, *C*, *D* are the correlation coefficients and the values are shown in Table 3.

**Table 3:** The value of the correlation coefficient in eqn (4)

Correlation coefficient	*A*	*B*	*C*	*D*
Value	−4.3573	621.26	1.8846	−281.39

### 3.2 Core flooding experiments

The core flooding experiments were conducted to determine the best oil recovery rate under different experimental conditions (as shown in Table 4). The temperature was fixed at 25 °C for all experiments.

**Table 4:** The experimental conditions of the core flooding experiments

Core no.	Permeability (mD)	Porosity (%)	Average pore throat diameter (μm)	Oil viscosity (mPa s)	Concentration of nanogel particles (wt%)
1	25	0.22	1.91	25	1.5
2	25	0.20	2.00	25	0.5
3	25	0.21	1.95	100	1.5
4	25	0.23	1.87	100	0.5
5	2500	0.23	18.65	25	1.5
6	2500	0.21	19.52	25	0.5
7	2500	0.24	18.26	100	1.5
8	2500	0.23	18.65	100	0.5

Eqn (5) was introduced to calculate the average diameter of pores and throats while the value of *τ* was 2.5
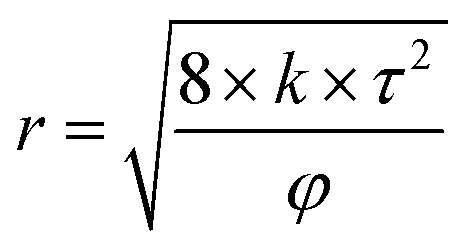
where *r* is the average diameter of pores and throats, μm; *k* is the permeability of the cores, μm^2^; *τ* is the tortuosity; *φ* is the porosity of the cores.

**Fig. 6:**
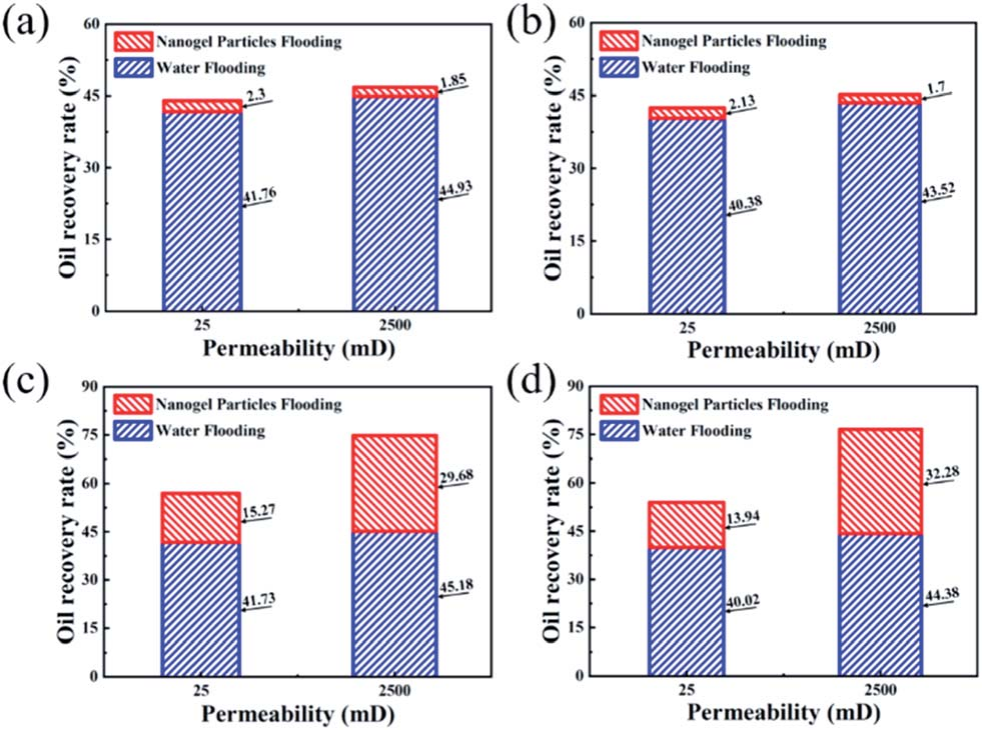
Oil recovery rates of core flooding experiments *versus* experimental conditions. (a) 25 mPa s oil and 0.5 wt% nanogel particles; (b) 100 mPa s oil and 0.5 wt% nanogel particles; (c) 25 mPa s oil and 1.5 wt% nanogel particles; (d) 100 mPa s oil and 1.5 wt% nanogel particles.

The experimental results of core flooding are shown in Fig. 6. In Fig. 6(a) and (b), the values of the oil recovery rate after 0.5 wt% nanogel particle dispersion flooding were almost same, although the other experimental conditions such as permeability and oil viscosity were different. In comparison with the 0.5 wt% nanogel particle dispersion, the 1.5 wt% nanogel particle dispersion had a significant incremental oil recovery after nanogel particle dispersion flooding. Fig. 6(c) shows that the oil recovery rates for the 25 mD and 2500 mD models were 15.27% and 29.68%, respectively, with the oil viscosity of 25 mPa s. Fig. 6(d) indicates that the oil recovery rates for 25 mD and 2500 mD were 13.94% and 32.28%, respectively, with the oil viscosity of 100 mPa s. Based on the results of the core flooding experiments, we came to the following two conclusions: the high concentration of nanogel particles was more conducive to enhanced oil recovery; nanogel particles have the potential for application in 2500 mD cores as compared with 25 mD cores.

### 3.3 Flow behaviors of nanogel particles

In order to analyze the flow behaviors of nanogel particles, the 2-D visualized model experiments were carried out to observe and analyze the characteristics of nanogel particles in porous media. Several parameters, including permeability, the concentration of the nanogel particles, injection direction and injection rate, were studied in depth.

#### 3.3.1 Effect of permeability

**Fig. 7:**
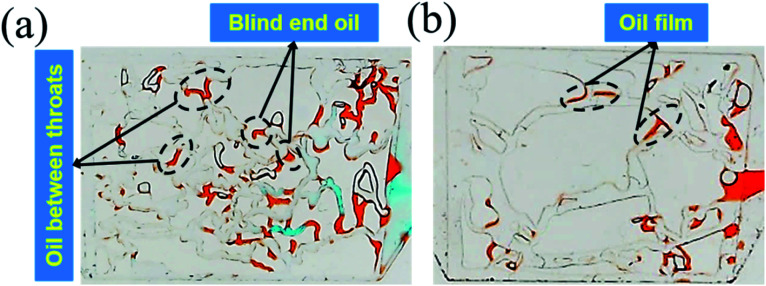
(a) The remaining oil distribution in the 25 mD model; (b) the remaining oil distribution in the 2500 mD model.

The effect of permeability on the remaining oil distribution is shown in Fig. 7. It can be seen that the distribution of the remaining oil was totally different in different permeability models after nanogel particle flooding. The concentration of nanogel particles was 1.5 wt% and the injection rate was 5 μL min^−1^. Fig. 7(a) shows that the types of remaining oil were classified into blind end oil and oil between throats, while Fig. 7(b) suggests that the type of remaining oil was oil film after nanogel particle flooding. Moreover, compared to the 2500 mD model (Fig. 7(b)), more remaining oil was trapped in pores and throats in the 25 mD model (Fig. 7(a)). These experimental results are consistent with the core flooding results (as shown in Fig. 6).

The above results were attributed to the different structures and sizes of pores and throats in different permeability models. As seen in Fig. 7(a), the tortuosity and coordination number of the pore throat in 25 mD were higher as compared to that in 2500 mD, causing more blind end holes and trapping more remaining oil. Moreover, eqn (5) implies that smaller diameters of pores and throats existed in 25 mD. Thus, the capillary force calculated according to eqn (6) in the 25 mD model was higher than that in the 2500 mD model. The wettability of the surface of the model was oil-wet, thus the capillary force was resistant to the oil displacement, which leads to more remaining oil being trapped in the 25 mD model; the higher the permeability, the larger the pore size. Therefore, the majority of pores and throats could be swept by the nanogel particles in the 2500 mD model. Combined with the oil displacement effect of the nanogel particles, little remaining oil was left in the 2500 mD model (Fig. 7(b)). As a result, the greater oil recovery rate was obtained at the permeability of the 2500 mD core.

#### 3.3.2 Effect of concentration of nanogel particles

**Fig. 8:**
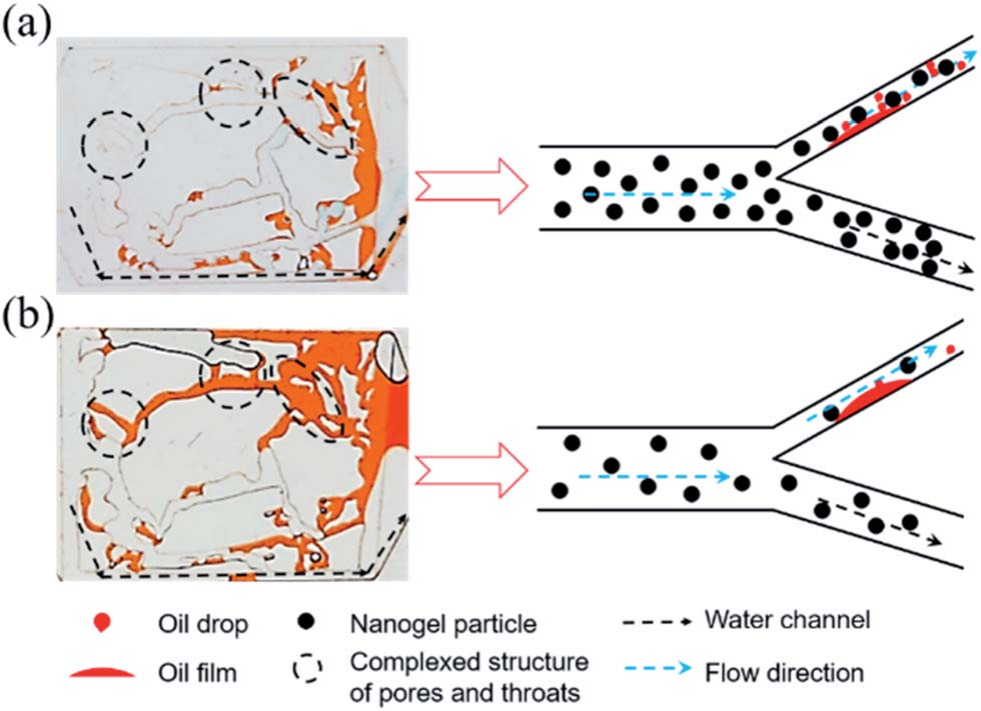
(a) Distribution of the remaining oil after 1.5 wt% nanogel particle flooding; (b) distribution of the remaining oil after 0.5 wt% nanogel particle flooding.

Fig. 8 shows the effect of the nanogel concentration on the flow behaviors and remaining oil distribution. It can be seen that the quantities of the remaining oil were distinctly different in Fig. 8(a) and (b). The injection rate was 5 μL min^−1^. As seen, more remaining oil was left in the porous media after 0.5 wt% nanogel particle flooding as compared with that after 1.5 wt% nanogel particle flooding. It is also worth noting that more remaining oil was distributed in the tops of the models for both 0.5 wt% and 1.5 wt% nanogel particles systems, which was attributed to the formation of water channels in the bottom of the models after water flooding. Thus, the nanogel particles had priority access to the water channel due to the smaller percolation resistance during the process of nanogel particle flooding. For this reason, one of the objectives for the nanogel particles was to increase the percolation resistance and enhance the sweep efficiency by plugging the water channels.^12,32,33^

The viscosity of the nanogel particle dispersion with the concentration of 0.5 wt% could be negligible (as shown in Fig. 5) as compared to the 1.5 wt% nanogel particle dispersion, which caused more remaining oil to be left as shown in Fig. 8(b). In Fig. 8(a), more nanogel particles preferred to flow through the water channels, causing a plugging wall and the increase in the percolation resistance. Thus, the subsequent nanogel particles were forced into the micron–nano scale pore throat, which increased the sweep efficiency and oil recovery rate. In terms of the 0.5 wt% nanogel particle dispersion, the plugging effect was not successful due to the presence of fewer nanogel particles. As a result, the nanogel particles only flowed along the water channel. Thus, the oil in the small pores and throats was not able to be effectively displaced during the process of nanogel particle flooding, as shown in Fig. 8(b). In addition, the greater viscosity of the nanogel particle dispersion with 1.5 wt% nanogel particles was also attributed to stronger intermolecular association among more nanogel particles, which formed three-dimensional network structures.^34^

#### 3.3.3 The effect of injection direction

**Fig. 9:**
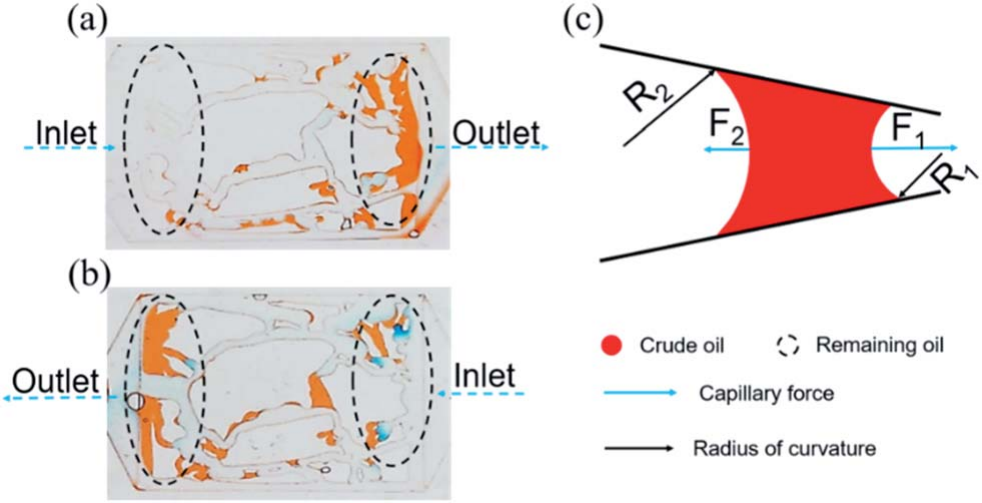
Distribution of the remaining oil in the injection direction (a) from left to right, (b) from right to left. (c) Force analysis of the curve interface.

Fig. 9 demonstrates that the distribution of the remaining oil was opposite in porous media when the injection direction was reversed with the injection rate of 5 μL min^−1^ at the concentration of 1.5 wt%. As seen in Fig. 9(a), a large amount of remaining oil was trapped near the outlet. Meanwhile, the majority of remaining oil also existed in the vicinity of the outlet (as shown in Fig. 9(b)) while the injection direction was opposite.

The above experimental results are attributed to the structures of the pores and throats in the porous media. In terms of the injection direction as shown in Fig. 9(a), the width of the pores and throats gradually decreased near the inlet as drawn in Fig. 9(c). There were two different meniscuses with different radii of curvature (*R*_1_ and *R*_2_) on both sides of the oil phase in throats. According to the Laplace equation as shown in eqn (6), the capillary force was calculated and recorded as *F*_1_ and *F*_2_:6
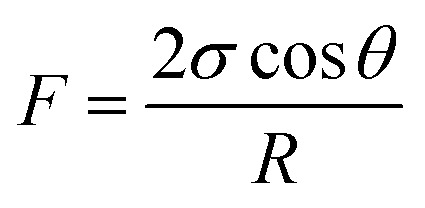
where *F* is the capillary force, Pa; *R* is the radius of the curvature of the meniscus, m; *σ* is the interfacial tension, N m^−1^; *θ* is the contact angle.

Obviously, *F*_1_ was larger than *F*_2_ due to the *R*_1_ < *R*_2_, representing the direction of the net force of the capillary force pointing to the right consistent with the displacement direction. Thus, the capillary force was beneficial for displacing oil near the inlet. On the contrary, the characteristics of the throat near the outlet were the opposite, leading to the net force of the capillary force pointing to the left, inconsistent with the displacement direction (as shown in Fig. 9(c)). Therefore, the capillary force was resistant to oil displacement, causing the remaining oil to be left mainly near the outlet. However, the direction of the net capillary force was opposite when the injection direction was the opposite. In other words, the capillary force played an opposite role at the same position in porous media. Thus, the distribution of the remaining oil was also in the vicinity of the outlet (as shown in Fig. 9(b)).

#### 3.3.4 Effect of injection rate

**Fig. 10:**
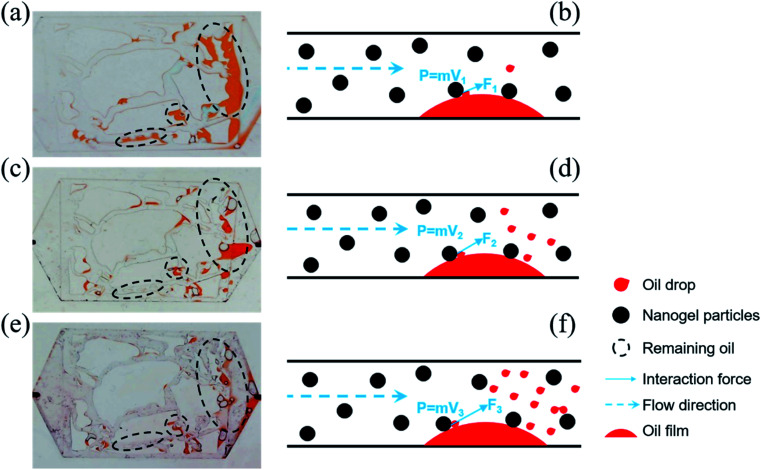
Distribution of the remaining oil at the injection rate of (a) 5 μL min^−1^ (*V*_1_), (c) 20 μL min^−1^ (*V*_2_), (e) 50 μL min^−1^ (*V*_3_). (b, d and f) The respective mechanisms of various injection rates on the initiating oil.

Fig. 10 implies that the amount of remaining oil decreased with the increase in the injection rate. The concentration of nanogel particles was 1.5 wt%. Three types of remaining oil containing oil film, blind end oil and oil between pores and throats, can be seen in Fig. 10(a). Fig. 10(c) shows that the majority of the oil film and oil trapped between pores and throats disappeared at the injection rate of 20 μL min^−1^. There was a little oil in the porous media when the injection rate was 50 μL min^−1^ in Fig. 10(e). However, there have been few reports on the mechanisms of injection rate to oil displacement efficiency.

The oil was trapped in porous media due to intermolecular forces between oil molecules, and oil molecules and the solid particles, which prevented the movement of oil molecules from the solid. However, once the oil molecules enhanced the energy to neutralize the binding force, they were easily displaced. Inspired by this, the momentum conservation laws were introduced to explain the mechanism of oil displacement with the influence of the injection rate. The collision between nanogel particles and oil droplets was considered to be a perfect elastic collision and eqn (7) was utilized to briefly describe the momentum conservation law.

7 *Ft* = *MV*′ + *mv*′ = *MV* + *mv*

where *F* is the contact force, N; *t* is the contact time, s; *M* is the quality of the nanogel particle; *V*′ is the velocity of the nanogel particle after contact; *m* is the quality of the oil droplet; *v*′ is the velocity of the oil droplet after contact; *V* is the velocity of the nanogel particle before contact; *v* is the velocity of the oil droplet before contact, regarded as 0.

*F* was beneficial in promoting oil droplets getting rid of the constraint of intermolecular forces. Eqn (7) demonstrates that *F* is proportional to the velocity *V*. Thus, the value of *F* would be increased on increasing the injection rate of the nanogel particles. The larger the value of *F*, the more oil droplets are replaced. Finally, there was less remaining oil in the porous media as the injection rate increased (as shown in Fig. 10(b), (d) and (f)). On the other hand, the plugging was formed more easily when the injection rate was high, which could increase the sweep efficiency.

### 3.4 EOR mechanisms of nanogel particles

**Fig. 11:**
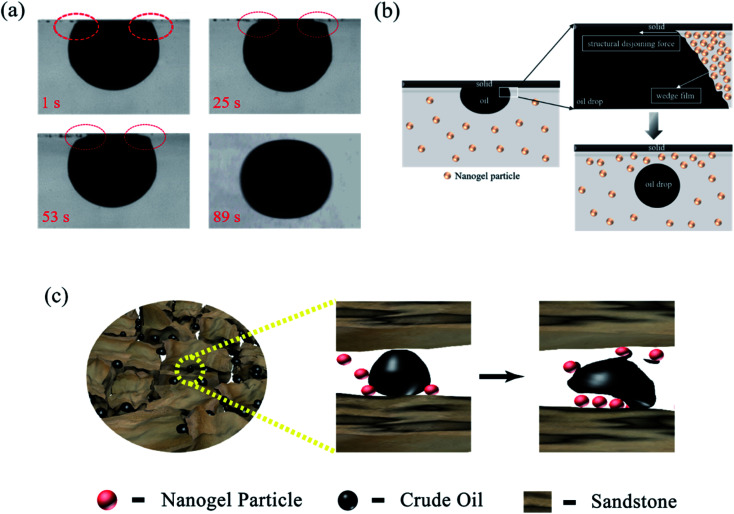
EOR mechanisms of nanogel particles. (a) Characteristics of oil-drop immersion in the nanogel particle dispersion *versus* time. (b) The process of the wedge film enhancing the spreading of the nanogel particles on the solid; (c) dynamic EOR mechanism of nanogel particles.

Fig. 11 revealed the EOR mechanism of the nanogel particles based on the immersion experiment and 2-D visualized experiments. The immersion experiment involved the immersion of one oil droplet, which was adhered to a solid, in the 1.5 wt% nanogel particle dispersion and measuring the contact angle. The 2-D visualized model experiments, including the effects of permeability, the concentration of the nanogel particles, injection direction and injection rate on the flow behaviors of nanogel particles in porous media, were also conducted.

Fig. 11(a) shows the characteristics of the oil drop immersion in the static nanogel particle dispersion *versus* time. It is worth noting that the oil droplet was gradually moving away from the solid at both ends of the three-phase contact zone. Interestingly, the oil droplet was completely detached from the solid after 89 seconds. The above result may be ascribed to the presence of a force at the three-phase contact zone. Fig. 2(c) indicates that the diameter of the nanogel particles was 30 nm, which would cause the spreading behavior along the solid. Thus, the phenomenon (as shown in Fig. 11(a)) was attributed to the osmotic pressure, which caused the wedge film along its interface among the solid, nanogel particles and oil droplets.^35,36^ The structure of the nanogel particles within the wedge film could enhance the spreading of the nanogel particles on the solid. This would indicate that the wedge film induced an additional pressure force called the structural disjoining pressure at its interface to remove the oil droplets from the solid into the displacement phase. The structural disjoining pressure increased evenly as the nanogel particles spread further (as shown in Fig. 11(b)).^37^

We also determined some other oil displacement mechanisms of nanogel particles from dynamic experiments (as shown in Fig. 11(c)). Firstly, the viscosity of the 1.5 wt% nanogel particle dispersion was 250 mPa s of oil viscosity at 90 °C, attributed to the stronger intermolecular force among nanogel particles, causing greater mobility control. Secondly, more energy was transferred to the oil droplets from the nanogel particles by collision according to the momentum conservation laws. The oil droplets then moved away from the solid by overcoming the adhesion forces.

## 4 Conclusion

High-viscosity nanogel particles with a diameter of 30 nm were synthesized through a free-radical reaction to combine the effects of the gel and nanoparticles. The high viscosity of the nanogel particle dispersion with 250 mPa s at 90 °C was utilized to plug water channels and drag oil droplets out of the remaining oil. Core flooding experiments also demonstrated that both light and heavy oil recovery rates reached around 30%. The EOR mechanisms and flow behaviors of nanogel particles were also studied. According to the 2-D visualized model experiments, the momentum conservation law was first introduced to interpret the effect of the injection rate on the oil displacement efficiency, which demonstrated that the higher the injection rate, the better the oil displacement efficiency. Moreover, the presence of the structural disjoining pressure at the three-phase contact zone was proven through an immersion experiment, which is beneficial for oil displacement. In conclusion, nanogel particle dispersions with high viscosity can increase the sweep efficiency and also improve oil displacement efficiency.

## Conflicts of interest

The authors declare no competing financial interest.

## Supplementary Material

## References

[cit1] Goudarzi A., Zhang H., Varavei A., Taksaudom P., Hu Y., Delshad M., Bai B., Sepehrnoori K. (2015). A Laboratory and Simulation Study of Preformed Particle Gels for Water Conformance Control. Fuel.

[cit2] Zhao S., Pu W., Wei B., Xu X. (2019). A Comprehensive Investigation of Polymer Microspheres (PMs) Migration in Porous Media: EOR Implication. Fuel.

[cit3] Rego F. B., Botechia V. E., Schiozer D. J. (2017). Heavy Oil Recovery by Polymer Flooding and Hot Water Injection Using Numerical Simulation. J. Pet. Sci. Eng..

[cit4] Bai B., Liu Y., Coste J.-P., Li L. (2007). Preformed Particle Gel for Conformance Control: Transport Mechanism Through Porous Media. SPE Reservoir Eval. Eng..

[cit5] Chauveteau G. , Omari A., Tabary R., Renard M. and Rose J., Controlling Gelation Time and Microgel Size for Water Shutoff, in SPE/DOE Improved Oil Recovery Symposium, April 3–5, Society of Petroleum Engineers, Tulsa, Oklahoma, 2000, SPE-59317-MS

[cit6] Cozic C., Rousseau D., Tabary R. (2009). Novel Insights into Microgel Systems for Water Control. SPE Prod. Oper..

[cit7] Pritchett J. , Frampton H., Brinkman J., Cheung S., Morgan J., Chang K., Williams D. and Goodgame G., Field Application of a New In-Depth Waterflood Conformance Improvement Tool, in SPE International Improved Oil Recovery Conference in Asia Pacific, October 20–21, Society of Petroleum Engineers, Kuala Lumpur, Malaysia, 2003, p. 8

[cit8] Qi Y.-B., Zheng C.-G., Lv C.-Y., Lun Z.-M., Ma T. (2018). Compatibility Between Weak Gel and Microorganisms in Weak Gel-Assisted Microbial Enhanced Oil Recovery. J. Biosci. Bioeng..

[cit9] Chauveteau G. , Tabary R., Renard M. and Omari A., Controlling In-Situ Gelation of Polyacrylamides by Zirconium for Water Shutoff, in SPE International Symposium on Oilfield Chemistry, February 16–19, Society of Petroleum Engineers, Houston, Texas, 1999, p. 9

[cit10] Coste J. P. , Liu Y., Bai B., Li Y., Shen P., Wang Z. and Zhu G., In-Depth Fluid Diversion by Pre-Gelled Particles. Laboratory Study and Pilot Testing, SPE/DOE Improved Oil Recovery Symposium, April 3–5, Society of Petroleum Engineers, Tulsa, Oklahoma, 2000, p. 8

[cit11] Al-Ibadi A., Civan F. (2013). Experimental Investigation and Correlation of Treatment in Weak and High-Permeability Formations by Use of Gel Particles. SPE Prod. Oper..

[cit12] Imqam A., Bai B. (2015). Optimizing the Strength and Size of Preformed Particle Gels for Better Conformance Control Treatment. Fuel.

[cit13] Sydansk R. D., Al-Dhafeeri A. M., Xiong Y., Seright R. S. (2004). Polymer Gels Formulated with a Combination of High- and Low-Molecular-Weight Polymers Provide Improved Performance for Water-Shutoff Treatments of Fractured Production Wells. SPE Prod. Facil..

[cit14] Alhuraishawy A. K., Sun X., Bai B., Wei M., Imqam A. (2018). Areal Sweep Efficiency Improvement by Integrating Preformed Particle Gel and Low Salinity Water Flooding in Fractured Reservoirs. Fuel.

[cit15] Chauveteau G. , Tabary R., Christel B., Renard M., Yujun F. and Omari A., In-Depth Permeability Control by Adsorption of Soft Size-Controlled Microgels, in SPE European Formation Damage Conference, May 13–14, Society of Petroleum Engineers, The Hague, Netherlands, 2003, p. 8

[cit16] Wu Y.-S. and Bai B., Modeling Particle Gel Propagation in Porous Media, in SPE Annual Technical Conference and Exhibition, September 21–24, Society of Petroleum Engineers, Denver, Colorado, USA, 2008, p. 10

[cit17] Zaitoun A. , Tabary R., Rousseau D., Pichery T. R., Nouyoux S., Mallo P. and Braun O., Using Microgels to Shut Off Water in a Gas Storage Well, in International Symposium on Oilfield Chemistry, February 28 – March 2, Society of Petroleum Engineers, Houston, Texas, U.S.A., 2007, p. 8

[cit18] Rousseau D. , Chauveteau G., Renard M., Tabary R., Zaitoun A., Mallo P., Braun O. and Omari A., Rheology and Transport in Porous Media of New Water Shutoff/Conformance Control Microgels, in SPE International Symposium on Oilfield Chemistry, February 2–4, Society of Petroleum Engineers, The Woodlands, Texas, 2005, p. 12

[cit19] Al-Anazi H. A. and Sharma M. M., Use of a pH Sensitive Polymer for Conformance Control, in International Symposium and Exhibition on Formation Damage Control, February 20–21, Society of Petroleum Engineers, Lafayette, Louisiana, 2002, p. 8

[cit20] Benson I. P. , Nghiem L. X., Bryant S. L. and Huh C., Development and Use of a Simulation Model for Mobility/Conformance Control Using a pH-Sensitive Polymer, in SPE Annual Technical Conference and Exhibition, November 11–14, Society of Petroleum Engineers, Anaheim, California, U.S.A., 2007, p. 10

[cit21] Lau H. C., Yu M., Nguyen Q. P. (2017). Nanotechnology for Oilfield Applications: Challenges and Impact. J. Pet. Sci. Eng..

[cit22] Binns C. , Introduction to Nanoscience and Nanotechnology, Wiley, New York, NY, 2010

[cit23] Cao G. , Nanostructures and Nanomaterials - Synthesis, Properties, and Applications, Imperial College Press, London, 2004

[cit24] Binks B. (2002). Particles as Surfactants—Similarities and Differences. Curr. Opin. Colloid Interface Sci..

[cit25] Aveyard R., Binks B. P., Clint J. H. (2003). Emulsions Stabilised Solely by Colloidal Particles. Adv. Colloid Interface Sci..

[cit26] Hunter T. N., Wanless E. J., Jameson G. J., Pugh R. J. (2009). Non-Ionic Surfactant Interactions with Hydrophobic Nanoparticles: Impact on Foam Stability. Colloids Surf., A.

[cit27] Moghadam T. F., Azizian S. (2014). Effect of ZnO Nanoparticle and Hexadecyltrimethylammonium Bromide on the Dynamic and Equilibrium Oil–Water Interfacial Tension. J. Phys. Chem. B.

[cit28] Ravera F., Ferrari M., Liggieri L., Loglio G., Santini E., Zanobini A. (2008). Liquid–Liquid Interfacial Properties of Mixed Nanoparticle–Surfactant Systems. Colloids Surf., A.

[cit29] Ma H., Luo M., Dai L. L. (2008). Influences of Surfactant and Nanoparticle Assembly on Effective Interfacial Tensions. Phys. Chem. Chem. Phys..

[cit30] Bai B., Li L., Liu Y., Liu H., Wang Z., You C. (2007). Preformed Particle Gel for Conformance Control: Factors Affecting Its Properties and Applications. SPE Reservoir Eval. Eng..

[cit31] Sang Q., Li Y., Yu L., Li Z., Dong M. (2014). Enhanced Oil Recovery by Branched-Preformed Particle Gel Injection in Parallel-Sandpack Models. Fuel.

[cit32] Saghafi H. R. (2018). Retention Characteristics of Enhanced Preformed Particle Gels (PPGs) in Porous Media: Conformance Control Implications. J. Pet. Sci. Eng..

[cit33] Goudarzi A., Almohsin A., Varavei A., Taksaudom P., Hosseini S. A., Delshad M., Bai B., Sepehrnoori K. (2017). New Laboratory Study and Transport Model Implementation of Microgels for Conformance and Mobility Control Purposes. Fuel.

[cit34] Clarke A., Howe A., Mitchell J., Staniland J., Hawkes L. (2016). How Viscoelastic-Polymer Flooding Enhances Displacement Efficiency. SPE.

[cit35] Lim S., Zhang H., Wu P., Nikolov A., Wasan D. (2016). The Dynamic Spreading of Nanofluids on Solid Surfaces – Role of the Nanofilm Structural Disjoining Pressure. J. Colloid Interface Sci..

[cit36] Wasan D. T., Nikolov A. D. (2003). Spreading of Nanofluids on Solids. Nature.

[cit37] Lim S., Wasan D. (2017). Structural Disjoining Pressure Induced Solid Particle Removal from Solid Substrates Using Nanofluids. J. Colloid Interface Sci..

